# Effects of Landscape Compositional Heterogeneity and Spatial Autocorrelation on Environmental Niche and Dispersal in Simulated Organisms

**DOI:** 10.1002/ece3.71638

**Published:** 2025-07-13

**Authors:** Joseph Tardanico, Thomas Hovestadt

**Affiliations:** ^1^ Department of Animal Ecology & Tropical Biology Julius‐Maximilians Universität Würzburg Germany

## Abstract

Local adaptation, environmental tolerance, and dispersal mutually influence the evolution of one another and each are in turn influenced by landscape spatial structure. While each of the three have been investigated frequently in isolation in relation to spatial structure, the three have rarely been considered together. In this study, we explored how the magnitude of landscape environmental heterogeneity (compositional heterogeneity), and environmental spatial autocorrelation jointly affect the evolution of environmental niche optima, tolerance, dispersal frequency, and dispersal distance using a spatially explicit individual based model simulating organisms living, reproducing, and dispersing within grid‐based fractal landscapes. Compositional heterogeneity tended to have the strongest influence over patterns while spatial autocorrelation typically played a mediating role. We found that niche adaptation and dispersal patterns were driven by a balance between pressure to avoid risk imposed by spatial heterogeneity and pressure to hedge against risk imposed by temporal environmental fluctuations. Dispersal frequency and dispersal distance were affected differently by spatial structure, underscoring the importance of considering the two independently.

## Graphical abstract

This study examines how local adaptation, environmental tolerance, and dispersal evolve in response to landscape spatial structure, specifically focusing on environmental heterogeneity and spatial autocorrelation. Using a spatially explicit individual‐based model, we found that compositional heterogeneity had the strongest influence, while spatial autocorrelation played a mediating role in selection on traits. Niche adaptation and dispersal patterns were shaped by the balance between avoiding spatial risks and hedging against temporal fluctuations, with dispersal frequency and distance affected differently by the landscape structure.

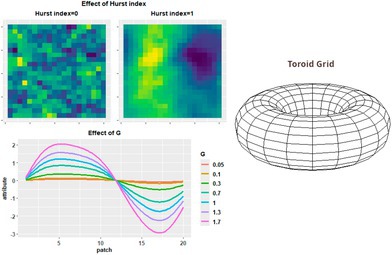

## 1 Introduction

Organisms exist within environments which vary over both time and space. Organisms can cope with spatial variation in their environments by adapting their niche optima to local conditions. Given adequate genetic variation in the population, such adaptation can occur quickly. However, successful adaptation requires organisms to cope with short‐term temporal variation in the environment (Hoffmann and Sgrò 2011). Organisms can deal with temporally variable environments by adapting their tolerances to local temporal variance. Alternatively, organisms can avoid periods of unfavorable conditions or hedge reproductive bets via dispersal (Venable and Brown 1988; Kisdi 2002), reducing the need to tolerate temporal variance in the environment (Bonte et al. 2012). However, because dispersal requires organisms to move through space and settle in new habitats, organisms relying on dispersal to avoid temporal environmental variance must be sufficiently capable of tolerating the spatial heterogeneity they encounter in their environments (Futuyma and Moreno 1988; Bonte et al. 2012). Tolerance to this spatial variation may not necessarily be conferred by tolerance to temporal variation as the environmental factors that vary over space may be different from those that vary over time. Since tolerances are critical to the ability of organisms both to stay in place and disperse, both strategies are subject to constraints (Bonte et al. 2012; Hillaert et al. 2015) imposed by trade‐offs between tolerance to environmental variation and performance under optimal conditions (Morin and Chuine 2006; Ravigné et al. 2009; Herren and Baym 2022), or trade‐offs between tolerances to variation in different environmental factors.

The evolution of local adaptation and environmental tolerances itself is affected by an organism's dispersal behavior through its effects on immigration and gene flow (Kirkpatrick and Barton 1997; Ronce and Kirkpatrick 2001; Lenormand 2002; Billiard and Lenormand 2005; Bridle et al. 2010, 2019). Dispersal and movement behavior, in turn, is informed by the risks imposed by spatial environmental heterogeneity and the selection it imposes, meaning that the evolution of local adaptation and environmental tolerances are dependent on spatial context (Bonte et al. 2006; Richardson et al. 2014; Forester et al. 2016), including the magnitude of compositional environmental heterogeneity and its spatial arrangement (Fahrig 2017). This dependence on spatial context has important implications for conservation, particularly in the face of climate change, as certain spatial structures may help or hinder local adaptation (Claudino and Campos 2014), range shifts (Burton et al. 2010; Synes et al. 2015; Årevall et al. 2018), and recolonization of habitat after disturbance (Leimar and Norberg 1997), making a thorough understanding of the effects of spatial structure on adaptation and dispersal key to creating effective conservation strategies (Holt and Barfield 2011; Årevall et al. 2018).

Studies investigating dispersal and adaptation commonly adopt a mechanistic modeling approach due to the large temporal and spatial scales such processes can occur over, the difficulty of observing them in nature, and the difficulty of experimentally manipulating conditions in the field (Hanski 2015; Ovaskainen et al. 2019). A mechanistic modeling approach has the advantage of allowing detailed experimental control over conditions while also enabling direct insight into causal mechanisms underpinning patterns by explicitly ecological and evolutionary processes (Cabral et al. 2017; Hanski 2015; Higgins et al. 2012). While numerous modeling studies have explored aspects of local adaptation (García‐Dorado 1987; Bridle et al. 2010; Claudino and Campos 2014; Kisdi et al. 2020), tolerance and niche breadth (Hillaert et al. 2015; Sieger et al. 2019; Kisdi et al. 2020), and dispersal (Hamilton and May 1977; Gros et al. 2006; Duputié and Massol 2013; Hillaert et al. 2015), few studies consider all three simultaneously. In their review of individual‐based models examining eco‐evolutionary dynamics, Romero‐Mujalli et al. (2019) found no studies which simultaneously modeled the evolution of local adaptation, dispersal, and phenotypic plasticity. Moreover, the authors also noted that studies focused on local adaptation were often not spatially explicit (e.g., Kisdi et al. 2020). Modeling studies on local adaptation which do consider spatial environmental variation tend to do so only in very simplified manners, often assuming simple linear gradients (e.g., Hillaert et al. 2015; Leidinger et al. 2021).

Meanwhile, studies modeling dispersal often explicitly consider spatial structure, but typically assume a binary habitat‐non‐habitat dichotomy (e.g., Gros et al. 2006; Claudino and Campos 2014). Such assumptions are problematic, particularly for terrestrial environments, as environmental shifts in space are often gradual, and many species exploit multiple habitat types (Hein et al. 2003; Jules and Shahani 2003), meaning it may be more appropriate in many cases to model landscapes as fractal environmental gradients or habitat mosaics (Fischer and Lindenmayer 2006; Franklin and Lindenmayer 2009). Sieger and Hovestadt (2020) used continuous fractal landscapes to explore the effect of the ratio of temporal to spatial heterogeneity on the evolution of dispersal frequency using an individual‐based model which notably modeled niche optimum, tolerance, and dispersal together as evolving traits. While the authors considered the magnitude of variation in patch environments (compositional heterogeneity), they did not explore the effects of spatial configuration despite its importance as a component of environmental spatial structure Fahrig (2017). Moreover, the authors assumed only random global dispersal and did not consider how environmental heterogeneity could affect other components of dispersal strategy such as dispersal distance, which may be affected by spatial heterogeneity independently of dispersal frequency (Gros et al. 2006; Bonte et al. 2010).

In this study, we use the model of Tardanico and Hovestadt (2023), developed as an extension of the model of Sieger and Hovestadt (2020), in order to systematically explore the effects of landscape structure on adaptation and dispersal strategy of annual asexual organisms with varying environmental niches and dispersal probabilities living, reproducing, and competing in continuous fractal landscapes. We extended the model by considering both temporally static and temporally variable patch environment attributes as well as by permitting dispersing organisms to choose between random global or nearest neighbor dispersal strategies, thus incorporating dispersal distance explicitly into the model. We specifically ask how the magnitude of spatial environmental variation, or compositional heterogeneity, and spatial environmental autocorrelation jointly affect the evolution of environmental niche optima, tolerances to environmental variation, dispersal frequency, and preference for shorter or longer distance dispersal, including the evolution of syndromes in these traits. In addition to data on organism traits such as niche optima, tolerances, and dispersal behavior, our model also records information on organism lineages and thus may be used to explore diversity patterns, which we previously explored in Tardanico and Hovestadt (2023). This study, however, will restrict itself to dealing with patterns of adaptation in organism traits.

## 2 Methods

We used the model which we developed for our previous study (Tardanico and Hovestadt 2023). As we made no modifications to the simulation model from our previous study, the description of the model's properties and its mechanics has been recycled from Tardanico and Hovestadt (2023).

### 2.1 Landscape Properties

**FIGURE 1:**
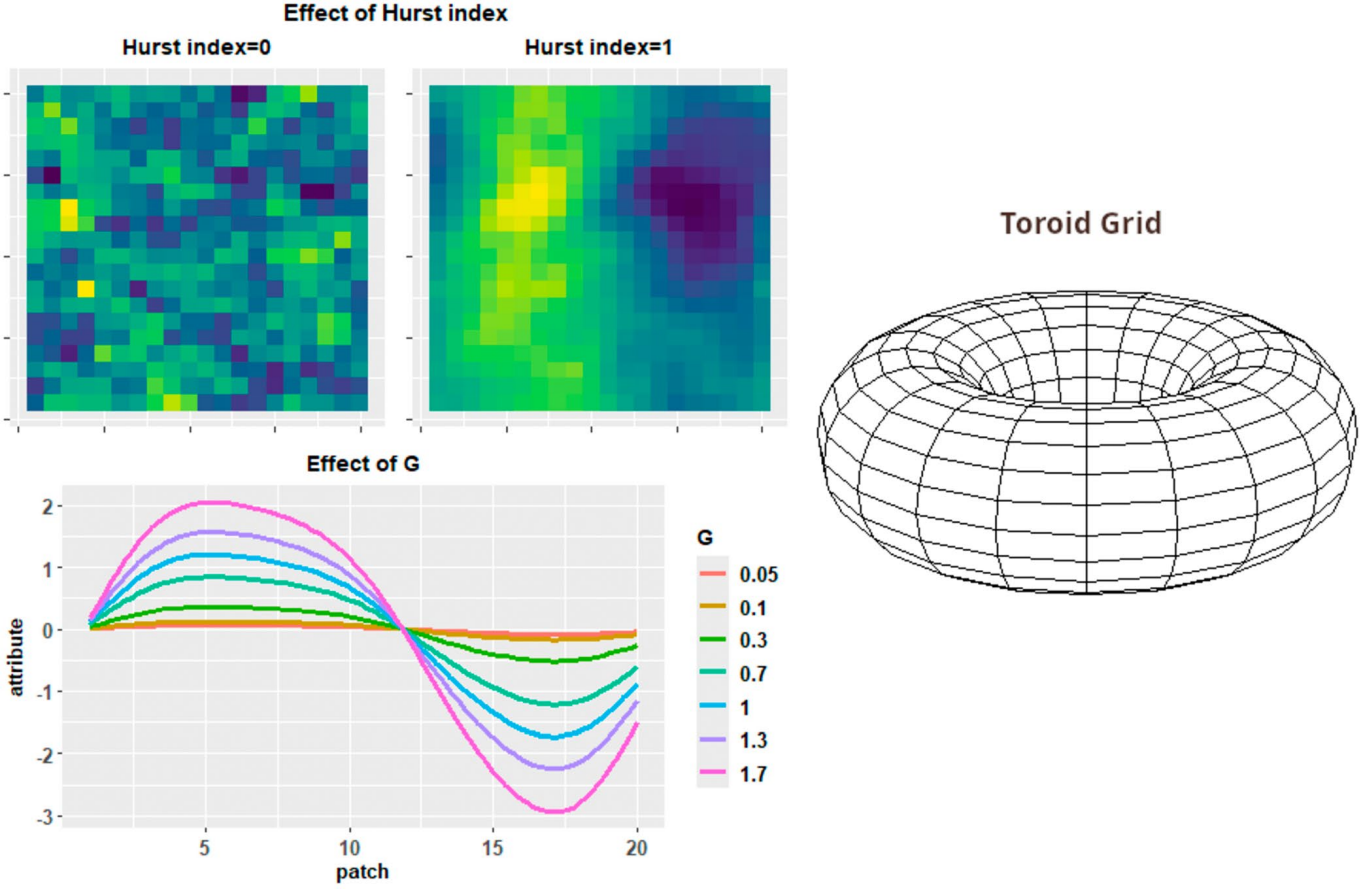
Diagram of major landscape properties. Top left: Effect of Hurst index on spatial autocorrelation. Bottom left: Effect of *G* on magnitude of variation in patch attributes. Right: Shape of simulated world. Landscapes are toroid with interconnected edges.

Landscapes consist of grids of habitat patches. Patches possess two attributes one representing patch temperature (*T*) and second attribute (*H*) representing an additional, unspecified environmental variable (e.g., a soil property). Spatial distributions for the two patch attributes were generated via an R implementation of the spatially autocorrelated landscape generation algorithm from Saupe (1988). This algorithm is capable of generating fractal landscapes with varying degrees of spatial autocorrelation between grid cell values depending on the value of the Hurst index parameter. Landscapes generated with this algorithm are toroid and opposite edges connect seamlessly to each other, thereby preventing edge effects from occurring at landscape edges (Figure 1). In this study, all landscapes were generated with a Hurst index of either 0 or 1. A Hurst index of 1 produces completely spatially autocorrelated landscapes where patches always have similar environments to their immediate neighbors, while a Hurst index of 0 produces a largely random spatial distribution of patch environments (Figure 1). Spatial distributions for the two patch attributes are generated independently, meaning that *T* and *H* attributes do not necessarily correlate with each other spatially. However, *T* and *H* spatial distributions for the same landscape were generated with matching generation parameters, including the Hurst index. Thus a landscape with a highly autocorrelated spatial distribution for the *T* attribute will always have an equally spatially autocorrelated *H* attribute distribution. Values for patch environmental attributes were drawn from a normal distribution and standardized to a mean of 0 and a standard deviation of 1, such that the average frequency of different patch environment values was constant regardless of spatial configuration. Landscape dimensions were set at 20 by 20 patches for a total of 400 patches in a landscape. These dimensions were chosen in order to limit computation time while still being large enough for structure driven patterns to emerge. Landscape compositional heterogeneity, the magnitude of spatial variation in the *T* and *H* attributes, was controlled by the simulation parameter *G*. By multiplying patch attribute values by *G*, the range of patch attribute values could be expanded or reduced while maintaining a constant configuration (Figure 1). In addition to varying spatially, the *T* attribute fluctuates over time such that the *T* attribute for patches varies from one time step to the next. Fluctuations in *T* are global and affect all patches in a landscape equally. Fluctuations in *T* are normally distributed with a mean of 0 and a standard deviation of 1 and modify patch *T* attributes by adding the value of the fluctuation to the patch's *T* attribute.

### 2.2 Organism Properties

Patches are inhabited by populations of asexual organisms belonging to lineages which behave as a guild of ecologically similar species who compete with each other within a patch. In addition to possessing a “taxonomic” identity, lineages possess varying environmental niches and dispersal tendencies, which serve to differentiate lineages functionally from one another. Organism niches are modeled as Gaussian curves whose center and spread are defined by a niche optimum and tolerance trait, respectively. Organisms possess separate optimum and tolerance traits for *T* and *H*. *T* niche optimum and tolerance are represented by the *T*_opt_ and *T*_tol_ traits, respectively, while *H* optimum and tolerance represented by the *H*_opt_ and *H*_tol_ traits. Organisms also possess two dispersal traits, $P_{\text{disp}}$, which defines the probability of an organism dispersing from its natal patch, and $P_{\text{global}}$ which defines an organism's preference for one of two possible dispersal modes. Dispersal is explained further in the section below. Organism traits are summarized in Table 1. Trait values are generated when a lineage first appears in a landscape by drawing random values from statistical distributions. Niche optima are drawn from a normal distribution with a μ of 0 and σ equal to *G*. Tolerance traits are drawn from a log‐normal distribution with a μ and σ of 0 and 1, respectively. Dispersal traits are drawn from a uniform distribution with a minimum of 0 and a maximum of 1. Organism traits are summarized in Table 1.

**TABLE 1:** Immigrant trait distributions and parameters. Modified from Tardanico and Hovestadt (2023).

Trait	Symbol	Distribution	Parameters
Temperature optimum	$T_{\mathrm{opt}}$	Normal	μ = $T_{\text{trend}}$, σ = *G*
Temperature tolerance	$T_{\mathrm{tol}}$	Log‐normal	μ = 0, σ = 1
Habitat optimum	$H_{\mathrm{opt}}$	Normal	μ = 0, σ = *G*
Habitat tolerance	$H_{\mathrm{tol}}$	Log‐normal	μ = 0, σ = 1
Dispersal chance	$P_{\text{disp}}$	Uniform	0, 1
Dispersal mode preference	$P_{\text{global}}$	Uniform	0, 1

### 2.3 Dispersal

Organisms can disperse from their natal patches to other patches. Individual organisms may disperse once during their life cycle. Whether or not an organism disperses from its natal patch is determined by drawing a random number from a uniform distribution and comparing the value with an organism's $P_{\text{disp}}$ trait. If the random number is less than or equal to the organism's $P_{\text{disp}}$ trait value, the organism will disperse. Dispersing organisms must then choose a dispersal mode. Two different modes of dispersal are possible within this model, serving as short and long distance modes. We chose to explicitly incorporate dispersal distance as a separate trait due to previous research indicating that landscape spatial structure affects dispersal distance differently from dispersal frequency (Gros et al. 2006). Organisms can disperse via nearest neighbor dispersal or random global dispersal. We chose these two dispersal methods because they are computationally lightweight, simple to implement, and already in widespread use in modeling studies (Travis and Dytham 1999; Kubisch et al. 2013, 2014; Kisdi et al. 2020). Used together, the two dispersal modes allow the model to approximate the peak and tail of a dispersal kernel without the complexity implementation and computational cost of directly simulating a dispersal kernel in a grid‐based landscape. The dispersal mode is selected by drawing a random number from a uniform distribution between 0 and 1 and comparing its value with an organism's $P_{\text{global}}$ trait. If the number's value is less than or equal to the organism's $P_{\text{global}}$ trait, the organism disperses via random global dispersal. If not, the organism disperses via nearest neighbor dispersal. In nearest neighbor dispersal, an organism moves to a random patch with the coordinates *x* + *p* and *y* + *q*, where *x* and *y* are the coordinates for the natal patch and *p* and *q* are integers between −1 and 1. If the target patch's coordinates are outside the bounds of the landscape, the organism is instead moved to the opposite side of the landscape due to the landscape being treated as a torus with interconnected edges, thereby avoiding the problem of artificially creating edge effects at landscape boundaries. In random global dispersal, a random patch within the landscape is selected as the target patch. In both dispersal modes, the target patch must have different coordinates from the natal patch and will be re‐selected if the target coordinates leave a dispersing organism in its natal patch.

### 2.4 Organism Life‐Cycle

Organisms have annual life cycles with complete replacement of the population at the end of a generation. Life cycles consist of discrete reproduction, competition, and dispersal phases. During the reproductive phase, organisms reproduce asexually to produce offspring with identical traits to their parents. The number of offspring is drawn from a Poisson distribution, with the expected reproductive output determined by an organism's fitness within its patch environment within a given time step as given by Equation (1). Here, $E_{\text{fert}}$ is the expected number of offspring, $R_{0}$ is an organism's intrinsic maximum expected offspring (kept at a constant value of 15), $T_{\text{patch}}$ and $H_{\text{patch}}$ are the temperature and habitat values for a given patch. Reproductive output is additionally limited by a trade‐off between tolerance and maximum expected offspring, meaning that organisms with broader tolerances produce fewer offspring on average. This trade‐off serves to prevent organisms from having infinitely large tolerances. The strength of this trade‐off is determined by the trade‐off parameter α (Chaianunporn and Hovestadt 2012; Sieger et al. 2019); lower values produce stronger trade‐offs. As the effect of varying α is functionally the same as the effect of varying the strength of *G*, α is kept at a constant value of 3 in this study. After reproduction, offspring undergo a maturation phase in which they compete on an equal basis with other offspring within the same patch. Survival of the competition phase is density dependent and regulated via the Beverton–Holt equations (Equations 2 and 3; Beverton and Holt 1957), where $S_{A}$ is the expected surviving offspring, $L_{0}$ is the total offspring, and *K* is the carrying capacity of a patch if all organisms in the patch have an $E_{\text{fert}}$ equal to $R_{0}$ and thus perfect fitness. Note that because patch carrying capacity is affected by $E_{\text{fert}}$, maladaptation may reduce the realized carrying capacity of a patch. The value of *K* is set at 150 individuals, which allows for relatively stable patch populations while maintaining low computation time. The number of surviving offspring are determined by drawing a random number from a binomial distribution with a mean of $S_{A}$. Surviving offspring are then able to disperse to a new patch and start the cycle anew.

(1) $$E_{\text{fert}}=R_{0}\cdot e^{\frac{-\left(T_{\text{patch}}-T_{\mathrm{opt}}\right)}{2T_{\mathrm{sd}}^{2}}}\cdot e^{\frac{-\left(H_{\text{patch}}-H_{\mathrm{opt}}\right)}{2H_{\mathrm{sd}}^{2}}}\cdot e^{\frac{-T_{\mathrm{sd}}^{2}}{2\alpha^{2}}}\cdot e^{\frac{-H_{\mathrm{sd}}^{2}}{2\alpha^{2}}}$$

(2) $$S_{A}=\frac{1}{1+a\cdot L_{0}}$$

(3) $$a=\frac{R_{0}-1}{K\cdot R_{0}}$$

### 2.5 Immigration From External Sources

New organisms can immigrate into the landscape from the outside. The number of new immigrants is randomly drawn from a Poisson distribution with an expected value of $E_{\text{immi}}$. In our simulations, $E_{\text{immi}}$ is set at a constant expected value of 2.5 immigrants per patch. This amounts on average to approximately 0.0011% of the expected local offspring production for a patch with a perfectly adapted population at carrying capacity. Immigrants are generated with randomized traits within a patch and added to the new generation along with existing offspring. Statistical distribution parameters for immigrant traits are summarized in Table 2.

**TABLE 2:** Summary of model parameters used in the experiment.

Parameter	Symbol	Value
Landscape dimensions		20 × 20 patches
Total simulation time‐steps	$t_{\max}$	10,000
Niche breadth trade‐off	α	3
Patch expected immigrants	$E_{\text{immi}}$	2.5
Gradient strength multiplier	*G*	∈0.05,0.1,0.3,0.7,1,1.3,1.7
Landscape Hurst Index	Hurst	∈0,1

### 2.6 Experiment Design

Landscapes were initialized from text files containing spatial distributions for the two patch attributes. Landscapes were initially empty with no preexisting populations and were then allowed to be colonized by immigrant organisms over the course of the simulation. Simulations were run for a total of 10,000 time steps. Simulations were run once for each landscape in a set for a total of 30 unique replicates. Fluctuations for each time step were generated at initialization. To ensure replicability, each replicate in a scenario was run with a unique, preset random number generator seed. We ran seven different compositional heterogneity scenarios (G∈0.05,0.1,0.3,0.7,1,1.3,1.7) and two spatial autocorrelation scenarios (Hurstindex∈0,1) for a total of 14 different scenarios. The simulation program recorded means and variances for trait values and fitness at each time step for entire landscapes, as well as a census of each individual organism in a landscape at the 10,000th timestep, including its lineage identity, trait values, and the patch it inhabited. The program then used the census data to calculate mean trait values and fitness for each patch in the landscape. We calculated two fitness metrics in this study, an organism's expected number of offspring, and the expected proportion of the maximum possible offspring. Model parameters used in this study are summarized in Table 2.

### 2.7 Data Analysis

We analyzed simulation output data in R (R Core Team 2020). Analysis was restricted to organisms belonging to lineages which had total landscape populations of 50 individuals or more at time step 10,000. We did this in order to restrict the analysis to lineages with established populations and exclude transient lineages with maladaptive trait combinations arising from the random nature of immigrant trait generation, as such lineages were unlikely to persist in the landscape beyond a few time steps. Due to the large size of the data set, we opted to sample 10,000 individuals from each scenario. In the case of the *G* = 1.3 scenarios, environmental fluctuations caused population crashes at the final time step, resulting in sample sizes of only 9742 (Hurst index = 0) and 9735 (Hurst index = 1) individuals, respectively. This resulted in a data set with a total of 139,477 observations. We assessed the data visually using the ggplot2 R package (Wickham 2016) and evaluated *R* ^2^ correlations between the six organism traits using the ggally package (Schloerke et al. 2024). We did not make use of statistical significance tests due to their lack of meaning within a mechanistic modeling context and their unreliability due to extreme sensitivity when sample sizes are extremely large (White et al. 2014).

## 3 Results

### 3.1 Organism Traits

**FIGURE 2:**
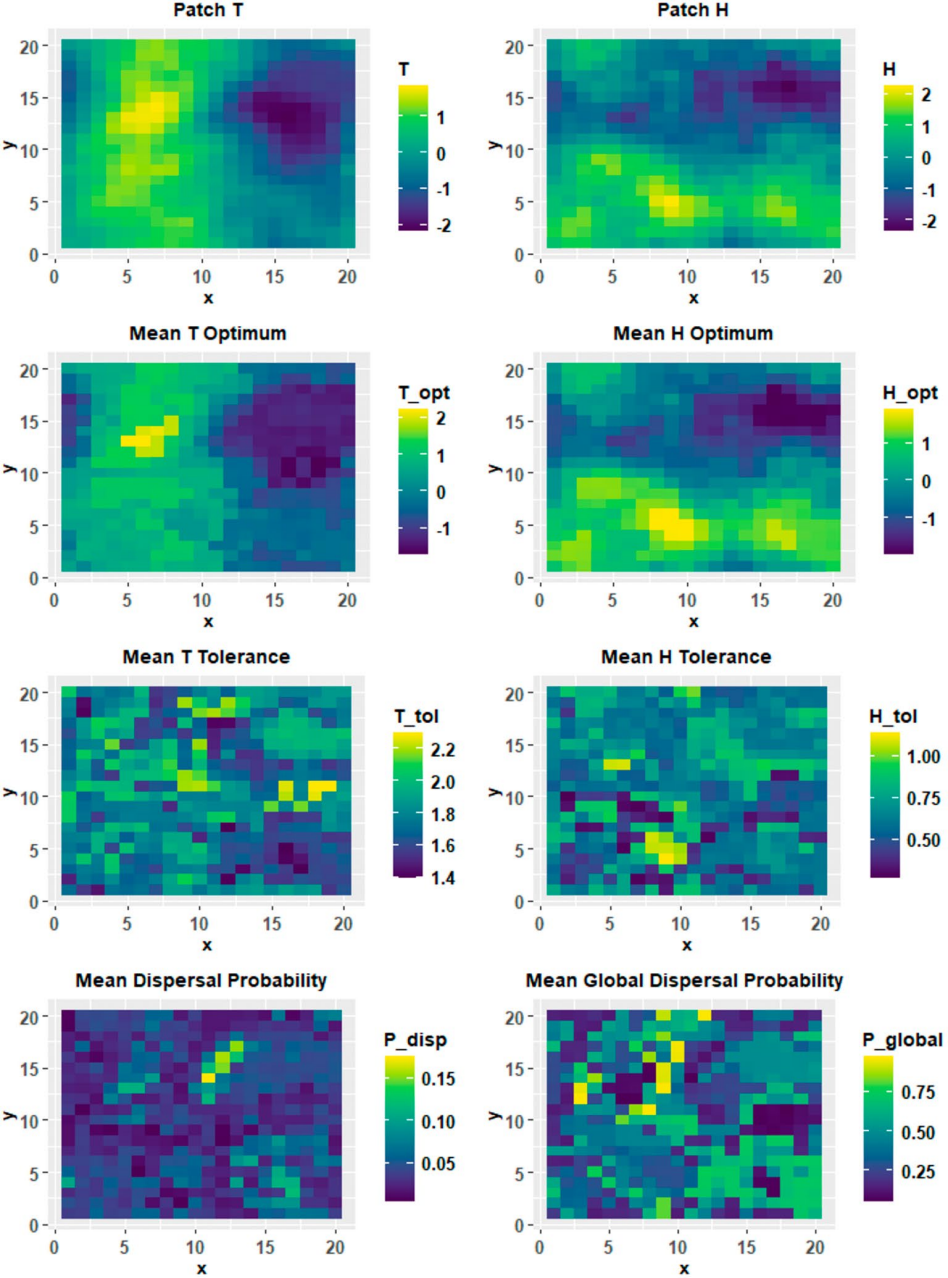
Example spatial maps of patch attributes and patch mean trait values for a single simulation replicate (*G* = 1, Hurst index = 1).

**FIGURE 3:**
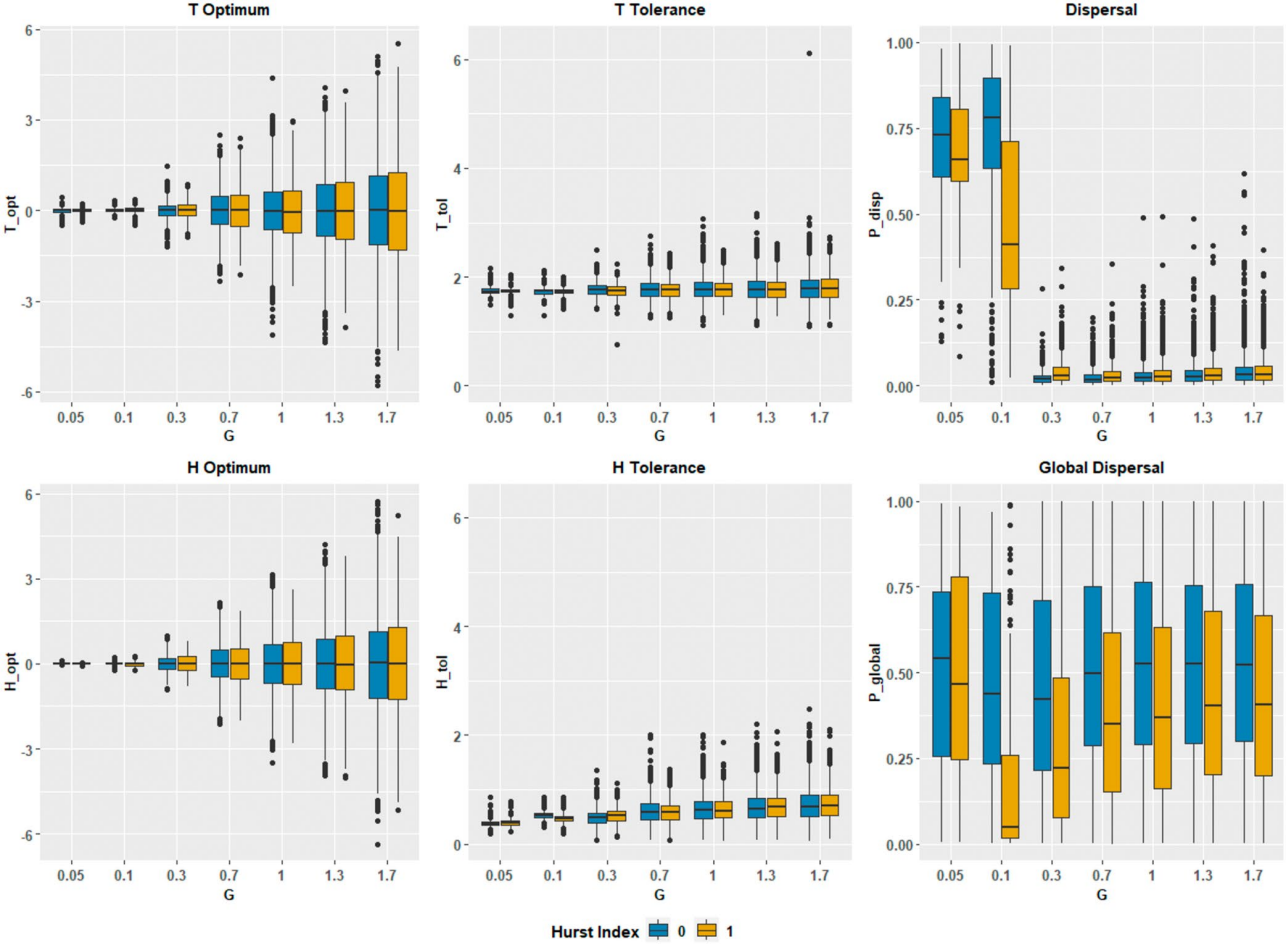
Box plot of distributions for organism trait values by *G* and Hurst index scenario at time step 10,000. *N* = 9742 (*G* = 1.3, Hurst index = 0), *N* = 9735 (*G* = 1.3, Hurst index = 1), *N* = 10,000 (all other scenarios).

Organism traits responded diversely to *G* and the Hurst index (Figures 2 and 3). Since these patterns were largely identical at the landscape level and when aggregated at the patch level, this subsection will focus on landscape level patterns. Niche optima traits $T_{\mathrm{opt}}$ and $H_{\mathrm{opt}}$ matched the frequency distributions for their respective patch attributes (Figures 2 and 3), with median values close to 0 and variances which increased with increasing *G*. Median *T* tolerance ($T_{\mathrm{tol}}$) showed little variation with *G*, but did increase in variance. $T_{\mathrm{tol}}$ was unaffected by the Hurst index. *H* tolerance ($H_{\mathrm{tol}}$) increased in both median and variance with greater *G*. This increase was monotonic under a Hurst index of 1, while under a Hurst index of 0 the increase was non‐monotonic between *G* = 0.05 and *G* = 0.3. Dispersal probability ($P_{\text{disp}}$) responded non‐monotonically to increasing *G*, shifting from high median values and relatively large variances to very low median values with small variances across a transition zone occurring between *G* = 0.05 and *G* = 0.3. This transition zone range was affected by the Hurst index, with the transition starting earlier and declining somewhat more mildly under a Hurst index of 1. From *G* = 0.3 onwards, $P_{\text{disp}}$ increased slightly with greater *G*. Within this range, $P_{\text{disp}}$ was slightly higher under a Hurst index of 1. Global dispersal probability ($P_{\text{global}}$) was highly variable in nearly all scenarios and responded non‐monotonically to increasing *G*, initially decreasing around *G* = 0.1 and then rebounding thereafter. This pattern was notably stronger under a Hurst index of 1. Median $P_{\text{global}}$ was consistently higher under a Hurst index of 0 (Figure 3).

### 3.2 Relationships Between Organism Traits

**FIGURE 4:**
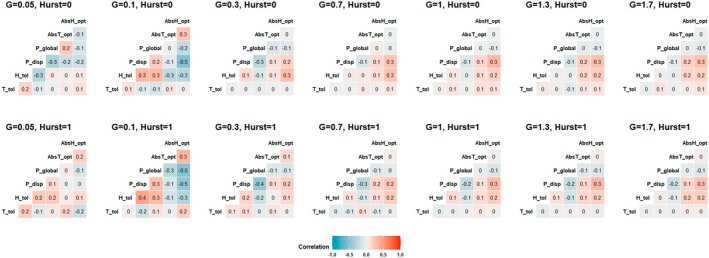
*R* ^2^ correlations between trait values by *G* and Hurst index scenario for individual organisms. Niche optima are absolute value transformed. *N* = 9742 (*G* = 1.3, Hurst index = 0), *N* = 9735 (*G* = 1.3, Hurst index = 1), *N* = 10,000 (all other scenarios).

Compositional heterogeneity and spatial autocorrelation affected the correlational relationships between organism traits (Figure 4). Compositional heterogeneity had the strongest effect on correlations between traits; spatial autocorrelation tended to mediate the strength of those correlations. In scenarios with a Hurst index of 1, trait correlations tended to be slightly stronger, although this effect was not universal.

Trait correlations shifted between three distinct patterns as *G* increased. The first pattern occurred at *G* = 0.05 and was characterized by strong but dramatically inconsistent relationships between traits. At *G* = 0.1, this pattern gave way to a pattern characterized by negative correlations between absolute value niche optima (*T*_opt_ and *H*_opt_), *P*_disp_ and positive correlations between *P*_disp_, *P*_global_, and *H*_tol_. A negative correlation between *P*_global_ and absolute value niche optima traits occurred under a Hurst index of 1 under this pattern, but not under a Hurst index of 0. Further increases in *G* resulted in a shift to a third pattern characterized by positive associations between absolute value niche optima, *P*_disp_, and *H*_tol_, and negative associations between *P*_disp_ and *P*_global_, and between *P*_global_ and absolute value *H*_opt_. Additionally, under a Hurst index of 1 there was a slight but consistent negative relationship between *P*_global_ and *H*_tol_ while the relationship between *P*_disp_ and *P*_global_ was slightly stronger. Associations were typically stronger with absolute value *H*_opt_ than *T*_opt_.

*T*_tol_ exhibited weak and inconsistent correlations with other traits across all scenarios, with a slightly higher tendency toward weak positive correlations with absolute value niche optima traits in Hurst index = 0 scenarios at or above *G* = 0.3. Correlations with *T*_tol_ tended to be strongest overall below *G* = 0.3; in *G* = 0.1 and *G* = 0.05 scenarios, *T*_tol_ had a consistent negative correlation with *P*_disp_.

### 3.3 Adaptation and Fitness

**FIGURE 5:**
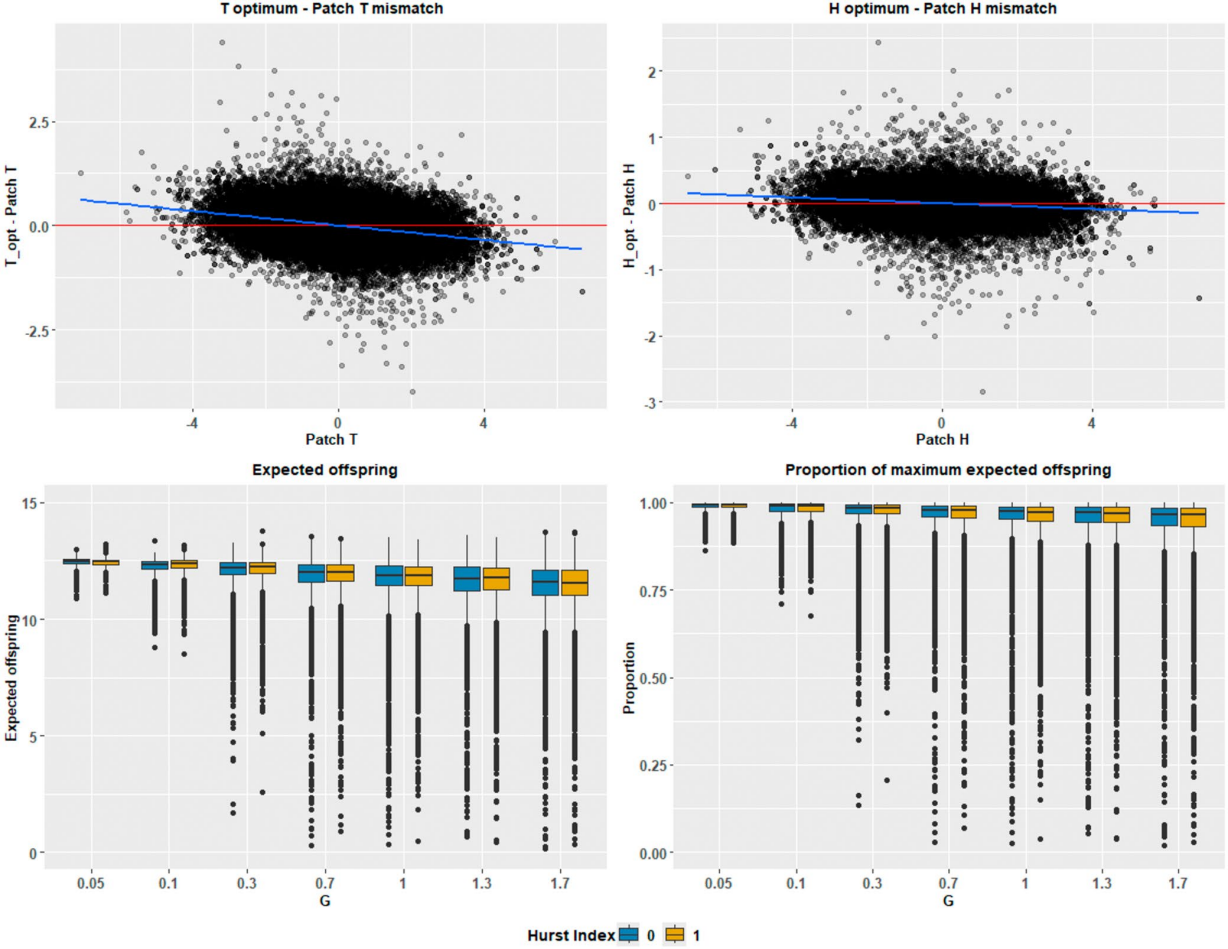
Individual local adaptation and fitness as measured by fertility. Top: Difference between individual niche optima and respective patch attributes versus patch attribute. Bottom: Box plots of distributions at time step 10,000 by scenario of individual expected offspring and the proportion of maximum expected offspring, the expected number of offspring under optimal conditions. *N* = 9742 (*G* = 1.3, Hurst index = 0), *N* = 9735 (*G* = 1.3, Hurst index = 1), *N* = 10,000 (all other scenarios).

Organisms were overall well adapted to their local patch conditions, with niche optima closely correlating with patch environment attributes. This correlation was strongest for *H*_opt_, with *T*_opt_ tending to be more variable in relation to patch *T* (Figure 5). Species inhabiting more extreme patches had a tendency toward greater mismatches between niche optima and patch attributes; this pattern was stronger for the *T* attribute than the *H* attribute.

Accordingly, fitness was relatively high, with the vast majority of organisms having at over 80% of their maximum fertility without accounting for reduced fertility due to the tolerance trade‐off. Factoring in the tolerance trade‐off, most individuals had expected at least 75% of their maximum fertility (Figure 3). Fertility declined slightly and increased in variance with increasing *G*.

## 4 Discussion

Landscape spatial structure affected niche traits and local adaptation, dispersal traits, and correlational relationships between traits. Patterns were most strongly influenced by compositional heterogeneity (*G*), with spatial autocorrelation (Hurst index) mostly playing a mediating role. Dispersal traits were an exception to this and were notably affected by both compositional heterogeneity and spatial autocorrelation. Niche optima distributions reflected the distributions of patch environment attributes in the landscapes and organisms were typically well adapted to their local patches, in line with theoretical studies findings that greater spatial heterogeneity selects for local adaptation due to the risk imposed by the landscape of immigrating into an unsuitable habitat (Hastings 1983; Futuyma and Moreno 1988). Tolerance traits behaved differently for the two environmental attributes. *T* tolerance was notably higher than habitat tolerance and was weakly affected by landscape structure, resembling the results of Sieger and Hovestadt (2020), and lacked any consistent relationships with other traits under most scenarios. *H* tolerance, on the other hand, showed a clear relationship with landscape structure at both the patch and landscape level. The difference in behavior of the two tolerance traits indicates that tolerance is determined primarily by the degree to which environmental variation can be avoided. In this model, and for many climate related environmental variables, temporal fluctuations are both unpredictable and can occur synchronously over a large area and thus affect organisms independently of spatial context or dispersal capabilities. Such environmental variation selects for broad niches that permit consistent fitness over a large range of conditions (Lynch and Gabriel 1987; Futuyma and Moreno 1988; Devictor et al. 2008; Lin and Wiens 2017), potentially overriding effects of smaller scale spatial variation. Purely spatial environmental variation, on the other hand, can be avoided substantially by restricting movement and dispersal, allowing organisms to retain more specialized niches.

Dispersal trait responses to compositional heterogeneity and spatial autocorrelation were nonlinear in nature and were largely consistent with established literature regarding relationships between dispersal frequency, dispersal distance, and spatial heterogeneity (Burgess et al. 2016). Dispersal shifted from a pattern of high dispersal probabilities at low levels of compositional heterogeneity to very low dispersal probabilities once heterogeneity increased beyond a threshold range, with slight increases in dispersal occurring at very high levels of compositional heterogeneity. Global dispersal probability shifted from a maximum at very low compositional heterogeneity to a minimum around the threshold range at which dispersal probability shifted and rebounded thereafter. Frequent dispersal is expected under very low spatial heterogeneity because there is little spatial variation in fitness and thus little risk to dispersal, while kin competition imposes a positive selection on dispersal (Hamilton and May 1977; Nakajima and Kurihara 1994; Gandon 1999). Moreover, kin competition selects for longer dispersal distances as this allows organisms to minimize the chance of encountering kin in a destination patch (Hovestadt et al. 2001; Rousset and Gandon 2002), resulting in a preference for random global dispersal under very low heterogeneity. Increasing compositional heterogeneity beyond a certain threshold resulted in a decline in dispersal chance across a transition zone as compositional heterogeneity began to exceed the limits of tolerances and impose increasingly significant fitness costs to dispersal (Hastings 1983; Holt 1985). This threshold itself was dependent on spatial autocorrelation and selection on dispersal traits within the transition zone was strongly divergent between autocorrelation scenarios. In the *G* = 0.1 scenarios, high autocorrelation produced an extremely broad range of dispersal probabilities and the lowest global dispersal probabilities of any scenario, while dispersal trait distributions under low autocorrelation were similar to other low heterogeneity scenarios. The discrepancy between the two autocorrelation scenarios when *G* = 0.1 is the result of several factors. Under low autocorrelation, unpredictable spatial variation selects for higher *H* tolerance, reducing risks associated with dispersal and making organisms less sensitive to spatial variance in the environment. In contrast, the predictability of spatial variation in highly autocorrelated landscapes results in lower *H* tolerance and thus greater sensitivity to spatial context. As a consequence, dispersal in the high autocorrelation *G* = 0.1 scenarios is subject to a range of different selective pressures depending on an organism's environmental niche. Organisms adapted to common habitats, or those with broad tolerances face relatively low dispersal risks as suitable habitat is plentiful, while organisms with more narrow niches, or those adapted to rarer or more extreme environments face high risks when dispersing resulting in selective pressure toward lower dispersal probabilities. The predictability of spatial variation in high autocorrelation scenarios also strongly favors nearest neighbor dispersal over random global dispersal if dispersal is undirected, as it will almost always result in an organism landing in a suitable patch under moderate compositional heterogeneity, while random global dispersal carries significant risk of emigration into an unsuitable patch (Bonte et al. 2010). In the absence of spatial autocorrelation, there is no meaningful advantage to either dispersal method, causing global dispersal to behave as a largely neutral trait. At *G* = 0.3 and above, spatial heterogeneity strongly selects for low dispersal probabilities; dispersal probabilities in these scenarios were similar to those observed by Sieger and Hovestadt (2020). Increases in dispersal and global dispersal probabilities with further increases in *G* above 0.3 indicate increasing selection for bet‐hedging against temporal heterogeneity due to decreasing habitat area. Dispersal was slightly but consistently higher in highly autocorrelated scenarios at and above *G* = 0.3 while global dispersal probability was consistently lower. These findings are consistent with the results of Hovestadt et al. (2001), which also found that higher spatial autocorrelation favored increased local dispersal propensity and distance and disfavored global dispersal.

Patterns of correlation between traits responded in a nonlinear fashion, with abrupt shifts in patterns at two thresholds of compositional heterogeneity, one above *G* = 0.1 and another threshold below *G* = 0.1. Above *G* = 0.1, patterns were highly similar between scenarios, with absolute value niche optima showing consistent positive relationships with tolerances and dispersal chance, and a consistent negative relationship between dispersal chance and global dispersal that diminishes with greater heterogeneity. Meanwhile, patterns at *G* = 0.1 were characterized by positive associations between dispersal chance, global dispersal, and *H* tolerance, and negative associations between *H* tolerance and dispersal traits, and absolute value niche optima. These results mirror those of Sieger and Hovestadt (2020), which found a shift in the location of the most frequent dispersers from the most common habitats to rare patches with more extreme environments as spatial heterogeneity increased, driven by greater selection for bet‐hedging strategies in organisms living in extreme patches. Similarly, trait correlation patterns in our model above *G* = 0.1 are consistent with increasingly strong selection for bet‐hedging strategies as niche optima move further away from average landscape conditions. This is further supported by a pattern of lower fitness in organisms adapted to more extreme conditions and the tendency for such organisms to be adapted to slightly more average conditions than those they experienced in their habitat patches. These patterns appear to be consequences of the smaller habitat area available to organisms with more extreme niche optima. The small habitat area reduces the population sizes that can be supported and renders such organisms particularly vulnerable to temporal environmental fluctuations (Lande 1993; Hanski 1998; Hill and Caswell 1999), increasing the importance of risk spreading strategies for population persistence. The need for insurance against temporal fluctuations may partly explain the slight tendency of “regression toward the mean” (Sieger and Hovestadt 2020) for niche optima in extreme patches as this apparent maladaptation may potentially expand the number of patches an organism can survive in at any given time. Below *G* = 0.1, trait correlations became highly idiosyncratic and inconsistent. This is likely due to a combination of very low variance in niche traits, weak selection within the range of values they occupy, very weak selection on dispersal traits, and highly uneven landscape communities dominated by a small number of lineages, leading to correlational patterns which are highly influenced by stochasticity and priority effects.

This model makes a number of simplifying assumptions for ease of implementation, computation, and analysis which, if altered, could affect selection on traits and resulting trait patterns. Our model assumes that organisms are asexual with an annual life cycle with no overlapping generations and does not consider other life histories or reproductive strategies. Longer lifespans allow for multiple bouts of reproduction which can serve to hedge reproductive bets in the face of temporal by spreading reproduction out over time (Danforth 1999; Hopper 1999; Gremer and Venable 2014). Inclusion of competing annual semelparous organisms and perennial iteroparous organisms would likely produce a pattern of succession over the course of the simulation with annual organisms dominating in the early stages and being gradually replaced by perennial organisms as the simulation progresses. Longer lifespans may also have the effect of reducing population turnover resulting in slower shifts in community level trait patterns. Inclusion of overlapping generations, meanwhile, has the potential to alter selection on dispersal traits as a result of the effect of age structure on kinship competition (Ronce et al. 2000). Including dormancy would provide organisms, including organisms with annual, semelparous life cycles, with an alternative means of hedging against reproductive risk by serving as a kind of dispersal through time (Buoro and Carlson 2014). As dormancy and dispersal serve similar bet hedging functions, the addition of dormancy as a possible strategy would likely reduce dispersal frequency. Organisms in our model are limited to two dispersal modes, and dispersal is assumed to be both undirected and unaffected by an organism's local environment or fitness. Undirected, uninformed dispersal can incur a notable fitness cost due to the risk that an organism will emigrate to an unsuitable patch or at an inopportune time (Hastings 1983; Bonte et al. 2010), necessitating greater tolerance which comes at the cost of maximum expected reproductive output in this model. Informed and directed dispersal can greatly reduce dispersal risk related fitness costs, particularly for long distance dispersal and under strong or unpredictable spatial environmental variation (Lakovic et al. 2015; Sieger and Hovestadt 2021). Reducing these fitness costs would likely permit more frequent dispersal and a higher reproductive output due to reduced selection for high tolerance. Finally, interactions with other organisms could affect selective pressures on traits in a variety of complex ways (Chaianunporn and Hovestadt 2012, 2019), but this model only considers competition.

## 5 Conclusions

Our study systematically explored the role of compositional heterogeneity and spatial autocorrelation in shaping both adaptation to environmental conditions and dispersal behavior in a temporally variable environment, something which to our knowledge has not been systematically explored by previous studies. Our model reproduced a number of patterns observed in previous theoretical studies stemming from varying degrees of selective pressure imposed by the spatial and temporal environments. We found that niche adaptation and dispersal patterns were primarily driven by a balance between pressure to avoid risk imposed by spatial heterogeneity and pressure to hedge against risk imposed by large‐scale temporal environmental fluctuations. Compositional heterogeneity tended to have the strongest influence over patterns while spatial autocorrelation typically played a mediating role. We found that dispersal frequency and dispersal distance were affected differently by spatial structure, underscoring the need to consider the two independently. Future studies should explore alternative life‐history and dispersal scenarios, as well as explore how a shifting environment interacts with landscape spatial structure to influence patterns of adaptation and dispersal behavior.

## Author Contributions

**Joseph Tardanico:** conceptualization (equal), formal analysis (equal), investigation (lead), methodology (lead), visualization (lead), writing – original draft (lead). **Thomas Hovestadt:** conceptualization (equal), formal analysis (equal), funding acquisition (lead), writing – review and editing (lead).

## Conflicts of Interest

The authors declare no conflicts of interest.

## Supporting information


Figures S1–S5

## Data Availability

Simulation output data used in this manuscript as well as configuration files and shell scripts used to run the simulations and the R scripts used for analysis are archived on the Dryad Digital Repository: https://doi.org/10.5061/dryad.mgqnk997t (Reviewer link: https://datadryad.org/stash/share/ZqHBR2sSrV_LUXcpjI9IPOHRne_ajU9xX7ONWK6hrt4). Code for the simulation program used to generate the data is available from GitHub: https://github.com/jtardanico/TardanicoHovestadt2023_Landscapes.

## References

[ece371638-bib-0001] Årevall, J. , R. Early , A. Estrada , U. Wennergren , and A. C. Eklöf . 2018. “Conditions for Successful Range Shifts Under Climate Change: The Role of Species Dispersal and Landscape Configuration.” Diversity and Distributions 24, no. 11: 1598–1611.

[ece371638-bib-0074] Beverton, R. J. H. , and S. J. Holt . 1957. On the Dynamics of Exploited Fish Populations. Fishery Investigations Series II Volume XIX, Ministry of Agriculture, Fisheries and Food. Springer Dordrecht. 10.1007/978-94-011-2106-4.

[ece371638-bib-0002] Billiard, S. , and T. Lenormand . 2005. “Evolution of Migration Under Kin Selection and Local Adaptation.” Evolution 59, no. 1: 13–23. 15792223

[ece371638-bib-0003] Bonte, D. , J. V. Borre , L. Lens , and J.‐P. Maelfait . 2006. “Geographical Variation in Wolf Spider Dispersal Behaviour Is Related to Landscape Structure.” Animal Behaviour 72, no. 3: 655–662.

[ece371638-bib-0004] Bonte, D. , T. Hovestadt , and H.‐J. Poethke . 2010. “Evolution of Dispersal Polymorphism and Local Adaptation of Dispersal Distance in Spatially Structured Landscapes.” Oikos 119, no. 3: 560–566.

[ece371638-bib-0005] Bonte, D. , H. Van Dyck , J. M. Bullock , et al. 2012. “Costs of Dispersal.” Biological Reviews 87, no. 2: 290–312. 21929715 10.1111/j.1469-185X.2011.00201.x

[ece371638-bib-0006] Bridle, J. R. , M. Kawata , and R. K. Butlin . 2019. “Local Adaptation Stops Where Ecological Gradients Steepen or Are Interrupted.” Evolutionary Applications 12, no. 7: 1449–1462. 31417626 10.1111/eva.12789 PMC6691213

[ece371638-bib-0007] Bridle, J. R. , J. Polechová , M. Kawata , and R. K. Butlin . 2010. “Why Is Adaptation Prevented at Ecological Margins? New Insights From Individual‐Based Simulations.” Ecology Letters 13, no. 4: 485–494. 20455923 10.1111/j.1461-0248.2010.01442.x

[ece371638-bib-0008] Buoro, M. , and S. M. Carlson . 2014. “Life‐History Syndromes: Integrating Dispersal Through Space and Time.” Ecology Letters 17, no. 6: 756–767. 24690406 10.1111/ele.12275

[ece371638-bib-0009] Burgess, S. C. , M. L. Baskett , R. K. Grosberg , S. G. Morgan , and R. R. Strathmann . 2016. “When Is Dispersal for Dispersal? Unifying Marine and Terrestrial Perspectives.” Biological Reviews 91, no. 3: 867–882. 26118564 10.1111/brv.12198

[ece371638-bib-0010] Burton, O. J. , B. L. Phillips , and J. M. Travis . 2010. “Trade‐Offs and the Evolution of Life‐Histories During Range Expansion.” Ecology Letters 13, no. 10: 1210–1220. 20718846 10.1111/j.1461-0248.2010.01505.x

[ece371638-bib-0011] Cabral, J. S. , L. Valente , and F. Hartig . 2017. “Mechanistic Simulation Models in Macroecology and Biogeography: State‐of‐Art and Prospects.” Ecography 40, no. 2: 267–280.

[ece371638-bib-0012] Chaianunporn, T. , and T. Hovestadt . 2012. “Evolution of Dispersal in Metacommunities of Interacting Species.” Journal of Evolutionary Biology 25, no. 12: 2511–2525. 23020160 10.1111/j.1420-9101.2012.02620.x

[ece371638-bib-0013] Chaianunporn, T. , and T. Hovestadt . 2019. “Dispersal Evolution in Metacommunities of Tri‐Trophic Systems.” Ecological Modelling 395: 28–38.

[ece371638-bib-0014] Claudino, E. S. , and P. R. Campos . 2014. “Landscape Structure and the Speed of Adaptation.” Physics Letters, Section A 378, no. 36: 2664–2671.

[ece371638-bib-0015] Danforth, B. N. 1999. “Emergence Dynamics and Bet Hedging in a Desert Bee, *Perdita portalis* .” Proceedings of the Royal Society of London, Series B: Biological Sciences 266, no. 1432: 1985–1994.

[ece371638-bib-0016] Devictor, V. , R. Julliard , and F. Jiguet . 2008. “Distribution of Specialist and Generalist Species Along Spatial Gradients of Habitat Disturbance and Fragmentation.” Oikos 117, no. 4: 507–514.

[ece371638-bib-0017] Duputié, A. , and F. Massol . 2013. “An Empiricist's Guide to Theoretical Predictions on the Evolution of Dispersal.” Interface Focus 3, no. 6: 20130028. 24516715 10.1098/rsfs.2013.0028 PMC3915845

[ece371638-bib-0018] Fahrig, L. 2017. “Ecological Responses to Habitat Fragmentation Per Se.” Annual Review of Ecology, Evolution, and Systematics 48: 1–23.

[ece371638-bib-0019] Fischer, J. , and B. D. Lindenmayer . 2006. “Beyond Fragmentation: The Continuum Model for Fauna Research and Conservation in Human‐Modified Landscapes.” Oikos 112, no. 2: 473–480.

[ece371638-bib-0020] Forester, B. R. , M. R. Jones , S. Joost , E. L. Landguth , and J. R. Lasky . 2016. “Detecting Spatial Genetic Signatures of Local Adaptation in Heterogeneous Landscapes.” Molecular Ecology 25, no. 1: 104–120. 26576498 10.1111/mec.13476

[ece371638-bib-0021] Franklin, J. F. , and D. B. Lindenmayer . 2009. “Importance of Matrix Habitats in Maintaining Biological Diversity.” Proceedings of the National Academy of Sciences 106, no. 2: 349–350. 10.1073/pnas.0812016105 PMC2626705 19129497

[ece371638-bib-0022] Futuyma, D. J. , and G. Moreno . 1988. “The Evolution of Ecological Specialization.” Annual Review of Ecology and Systematics 19, no. 1: 207–233.

[ece371638-bib-0023] Gandon, S. 1999. “Kin Competition, the Cost of Inbreeding and the Evolution of Dispersal.” Journal of Theoretical Biology 200, no. 4: 345–364. 10525395 10.1006/jtbi.1999.0994

[ece371638-bib-0024] García‐Dorado, A. 1987. “Polymorphism From Environmental Heterogeneity: Some Features of Genetically Induced Niche Preference.” Theoretical Population Biology 32, no. 1: 66–75.

[ece371638-bib-0025] Gremer, J. R. , and D. L. Venable . 2014. “Bet Hedging in Desert Winter Annual Plants: Optimal Germination Strategies in a Variable Environment.” Ecology Letters 17, no. 3: 380–387. 24393387 10.1111/ele.12241

[ece371638-bib-0026] Gros, A. , H. Joachim Poethke , and T. Hovestadt . 2006. “Evolution of Local Adaptations in Dispersal Strategies.” Oikos 114, no. 3: 544–552.

[ece371638-bib-0027] Hamilton, W. D. , and R. M. May . 1977. “Dispersal in Stable Habitats.” Nature 269, no. 5629: 578–581.

[ece371638-bib-0028] Hanski, I. 1998. “Metapopulation Dynamics.” Nature 396, no. 6706: 41–49.

[ece371638-bib-0029] Hanski, I. 2015. “Habitat Fragmentation and Species Richness.” Journal of Biogeography 42, no. 5: 989–993.

[ece371638-bib-0030] Hastings, A. 1983. “Can Spatial Variation Alone Lead to Selection for Dispersal?” Theoretical Population Biology 24, no. 3: 244–251.

[ece371638-bib-0031] Hein, S. , J. Gombert , T. Hovestadt , and H. Poethke . 2003. “Movement Patterns of the Bush Cricket Platycleis Albopunctata in Different Types of Habitat: Matrix Is Not Always Matrix.” Ecological Entomology 28, no. 4: 432–438.

[ece371638-bib-0032] Herren, C. M. , and M. Baym . 2022. “Decreased Thermal Niche Breadth as a Trade‐Off of Antibiotic Resistance.” ISME Journal 16, no. 7: 1843–1852. 35422477 10.1038/s41396-022-01235-6 PMC9213455

[ece371638-bib-0033] Higgins, S. I. , R. B. O'Hara , and C. Römermann . 2012. “A Niche for Biology in Species Distribution Models.” Journal of Biogeography 39, no. 12: 2091–2095.

[ece371638-bib-0034] Hill, M. , and H. Caswell . 1999. “Habitat Fragmentation and Extinction Thresholds on Fractal Landscapes.” Ecology Letters 2, no. 2: 121–127.

[ece371638-bib-0035] Hillaert, J. , J. Boeye , R. Stoks , and D. Bonte . 2015. “The Evolution of Thermal Performance Can Constrain Dispersal During Range Shifting.” Journal of Biological Dynamics 9, no. 1: 317–335. 26406927 10.1080/17513758.2015.1078503

[ece371638-bib-0036] Hoffmann, A. A. , and C. M. Sgrò . 2011. “Climate Change and Evolutionary Adaptation.” Nature 470, no. 7335: 479–485. 21350480 10.1038/nature09670

[ece371638-bib-0037] Holt, R. D. 1985. “Population Dynamics in Two‐Patch Environments: Some Anomalous Consequences of an Optimal Habitat Distribution.” Theoretical Population Biology 28, no. 2: 181–208.

[ece371638-bib-0038] Holt, R. D. , and M. Barfield . 2011. “Theoretical Perspectives on the Statics and Dynamics of Species' Borders in Patchy Environments.” American Naturalist 178, no. S1: S6–S25. 10.1086/661784 PMC5014989 21956092

[ece371638-bib-0039] Hopper, K. R. 1999. “Risk‐Spreading and Bet‐Hedging in Insect Population Biology.” Annual Review of Entomology 44, no. 1: 535–560. 10.1146/annurev.ento.44.1.535 15012381

[ece371638-bib-0040] Hovestadt, T. , S. Messner , and J. P. Hans . 2001. “Evolution of Reduced Dispersal Mortality and ‘Fat‐Tailed’ Dispersal Kernels in Autocorrelated Landscapes.” Proceedings of the Royal Society of London, Series B: Biological Sciences 268, no. 1465: 385–391. 10.1098/rspb.2000.1379 PMC1088618 11270435

[ece371638-bib-0041] Jules, E. S. , and P. Shahani . 2003. “A Broader Ecological Context to Habitat Fragmentation: Why Matrix Habitat Is More Important Than We Thought.” Journal of Vegetation Science 14, no. 3: 459–464.

[ece371638-bib-0042] Kirkpatrick, M. , and N. H. Barton . 1997. “Evolution of a Species' Range.” American Naturalist 150, no. 1: 1–23. 10.1086/286054 18811273

[ece371638-bib-0043] Kisdi, É. 2002. “Dispersal: Risk Spreading Versus Local Adaptation.” American Naturalist 159, no. 6: 579–596. 10.1086/339989 18707383

[ece371638-bib-0044] Kisdi, É. , H. C. Weigang , and M. Gyllenberg . 2020. “The Evolution of Immigration Strategies Facilitates Niche Expansion by Divergent Adaptation in a Structured Metapopulation Model.” American Naturalist 195, no. 1: 1–15. 10.1086/706258 31868542

[ece371638-bib-0045] Kubisch, A. , T. Degen , T. Hovestadt , and H. J. Poethke . 2013. “Predicting Range Shifts Under Global Change: The Balance Between Local Adaptation and Dispersal.” Ecography 36, no. 8: 873–882.

[ece371638-bib-0046] Kubisch, A. , R. D. Holt , H.‐J. Poethke , and E. A. Fronhofer . 2014. “Where Am I and Why? Synthesizing Range Biology and the Eco‐Evolutionary Dynamics of Dispersal.” Oikos 123, no. 1: 5–22.

[ece371638-bib-0047] Lakovic, M. , H.‐J. Poethke , and T. Hovestadt . 2015. “Dispersal Timing: Emigration of Insects Living in Patchy Environments.” PLoS One 10, no. 7: e0128672. 26132493 10.1371/journal.pone.0128672 PMC4489195

[ece371638-bib-0048] Lande, R. 1993. “Risks of Population Extinction From Demographic and Environmental Stochasticity and Random Catastrophes.” American Naturalist 142, no. 6: 911–927. 10.1086/285580 29519140

[ece371638-bib-0049] Leidinger, L. , D. Vedder , and J. S. Cabral . 2021. “Temporal Environmental Variation May Impose Differential Selection on Both Genomic and Ecological Traits.” Oikos 130, no. 7: 1100–1115.

[ece371638-bib-0050] Leimar, O. , and U. Norberg . 1997. “Metapopulation Extinction and Genetic Variation in Dispersal‐Related Traits.” Oikos 80: 448–458.

[ece371638-bib-0051] Lenormand, T. 2002. “Gene Flow and the Limits to Natural Selection.” Trends in Ecology & Evolution 17, no. 4: 183–189.

[ece371638-bib-0052] Lin, L.‐H. , and J. J. Wiens . 2017. “Comparing Macroecological Patterns Across Continents: Evolution of Climatic Niche Breadth in Varanid Lizards.” Ecography 40, no. 8: 960–970.

[ece371638-bib-0053] Lynch, M. , and W. Gabriel . 1987. “Environmental Tolerance.” American Naturalist 129, no. 2: 283–303.

[ece371638-bib-0054] Morin, X. , and I. Chuine . 2006. “Niche Breadth, Competitive Strength and Range Size of Tree Species: A Trade‐Off Based Framework to Understand Species Distribution.” Ecology Letters 9, no. 2: 185–195. 16958884 10.1111/j.1461-0248.2005.00864.x

[ece371638-bib-0055] Nakajima, T. , and Y. Kurihara . 1994. “Evolutionary Changes of Dispersiveness in Experimental Bacterial Populations.” Oikos 69: 217–223.

[ece371638-bib-0056] Ovaskainen, O. , J. Rybicki , and N. Abrego . 2019. “What Can Observational Data Reveal About Metacommunity Processes?” Ecography 42, no. 11: 1877–1886.

[ece371638-bib-0057] R Core Team . 2020. R: A Language and Environment for Statistical Computing. R Foundation for Statistical Computing.

[ece371638-bib-0058] Ravigné, V. , U. Dieckmann , and I. Olivieri . 2009. “Live Where You Thrive: Joint Evolution of Habitat Choice and Local Adaptation Facilitates Specialization and Promotes Diversity.” American Naturalist 174, no. 4: E141–E169. 10.1086/605369 19737113

[ece371638-bib-0059] Richardson, J. L. , M. C. Urban , D. I. Bolnick , and D. K. Skelly . 2014. “Microgeographic Adaptation and the Spatial Scale of Evolution.” Trends in Ecology & Evolution 29, no. 3: 165–176. 24560373 10.1016/j.tree.2014.01.002

[ece371638-bib-0060] Romero‐Mujalli, D. , F. Jeltsch , and R. Tiedemann . 2019. “Individual‐Based Modeling of Eco‐Evolutionary Dynamics: State of the Art and Future Directions.” Regional Environmental Change 19: 1–12.

[ece371638-bib-0061] Ronce, O. , S. Gandon , and F. Rousset . 2000. “Kin Selection and Natal Dispersal in an Age‐Structured Population.” Theoretical Population Biology 58, no. 2: 143–159. 11042105 10.1006/tpbi.2000.1476

[ece371638-bib-0062] Ronce, O. , and M. Kirkpatrick . 2001. “When Sources Become Sinks: Migrational Meltdown in Heterogeneous Habitats.” Evolution 55, no. 8: 1520–1531. 11580012 10.1111/j.0014-3820.2001.tb00672.x

[ece371638-bib-0063] Rousset, F. , and S. Gandon . 2002. “Evolution of the Distribution of Dispersal Distance Under Distance‐Dependent Cost of Dispersal.” Journal of Evolutionary Biology 15, no. 4: 515–523.

[ece371638-bib-0075] Saupe, D. 1988. “Algorithms for random fractals.” In The Science of Fractal Images, edited by H. O. Peitgen and D. Saupe . Springer. 10.1007/978-1-4612-3784-6_2.

[ece371638-bib-0064] Schloerke, B. , D. Cook , J. Larmarange , et al. 2024. “GGally: Extension to ‘ggplot2’.” R Package Version 2.2.1. https://github.com/ggobi/ggally.

[ece371638-bib-0065] Sieger, C. S. , M. M. Cobben , and T. Hovestadt . 2019. “Environmental Change and Variability Influence Niche Evolution of Isolated Natural Populations.” Regional Environmental Change 19: 1999–2011.

[ece371638-bib-0066] Sieger, C. S. , and T. Hovestadt . 2020. “The Degree of Spatial Variation Relative to Temporal Variation Influences Evolution of Dispersal.” Oikos 129, no. 11: 1611–1622.

[ece371638-bib-0067] Sieger, C. S. , and T. Hovestadt . 2021. “The Effect of Landscape Structure on the Evolution of Two Alternative Dispersal Strategies.” Ecological Processes 10: 1–13.33425642

[ece371638-bib-0068] Synes, N. W. , K. Watts , S. C. Palmer , et al. 2015. “A Multi‐Species Modelling Approach to Examine the Impact of Alternative Climate Change Adaptation Strategies on Range Shifting Ability in a Fragmented Landscape.” Ecological Informatics 30: 222–229.

[ece371638-bib-0069] Tardanico, J. , and T. Hovestadt . 2023. “Effects of Compositional Heterogeneity and Spatial Autocorrelation on Richness and Diversity in Simulated Landscapes.” Ecology and Evolution 13, no. 12: e10810. 38094150 10.1002/ece3.10810 PMC10716673

[ece371638-bib-0070] Travis, J. M. , and C. Dytham . 1999. “Habitat Persistence, Habitat Availability and the Evolution of Dispersal.” Proceedings of the Royal Society of London, Series B: Biological Sciences 266, no. 1420: 723–728.

[ece371638-bib-0071] Venable, D. L. , and J. S. Brown . 1988. “The Selective Interactions of Dispersal, Dormancy, and Seed Size as Adaptations for Reducing Risk in Variable Environments.” American Naturalist 131, no. 3: 360–384.

[ece371638-bib-0072] White, J. W. , A. Rassweiler , J. F. Samhouri , A. C. Stier , and C. White . 2014. “Ecologists Should Not Use Statistical Significance Tests to Interpret Simulation Model Results.” Oikos 123, no. 4: 385–388.

[ece371638-bib-0073] Wickham, H. 2016. ggplot2: Elegant Graphics for Data Analysis. Springer‐Verlag.

