# Supplementary material for: Effects of Landscape Compositional Heterogeneity and Spatial Autocorrelation on Environmental Niche and Dispersal in Simulated Organisms
Source: Ecol Evol. 2025 Jul 13;15(7):e71638. doi: 10.1002/ece3.71638 (PMC12256203; doi:10.1002/ece3.71638)
Supplement: Supplementary file 1 — Figures S1–S5 [file ECE3-15-e71638-s001.pdf]

P_{disp} (G≥0.3) GAM and ANOVA

Fig. 1) Scatter plot of P_disp vs. G between G=0.3 and G=1.7 with GAM smoother

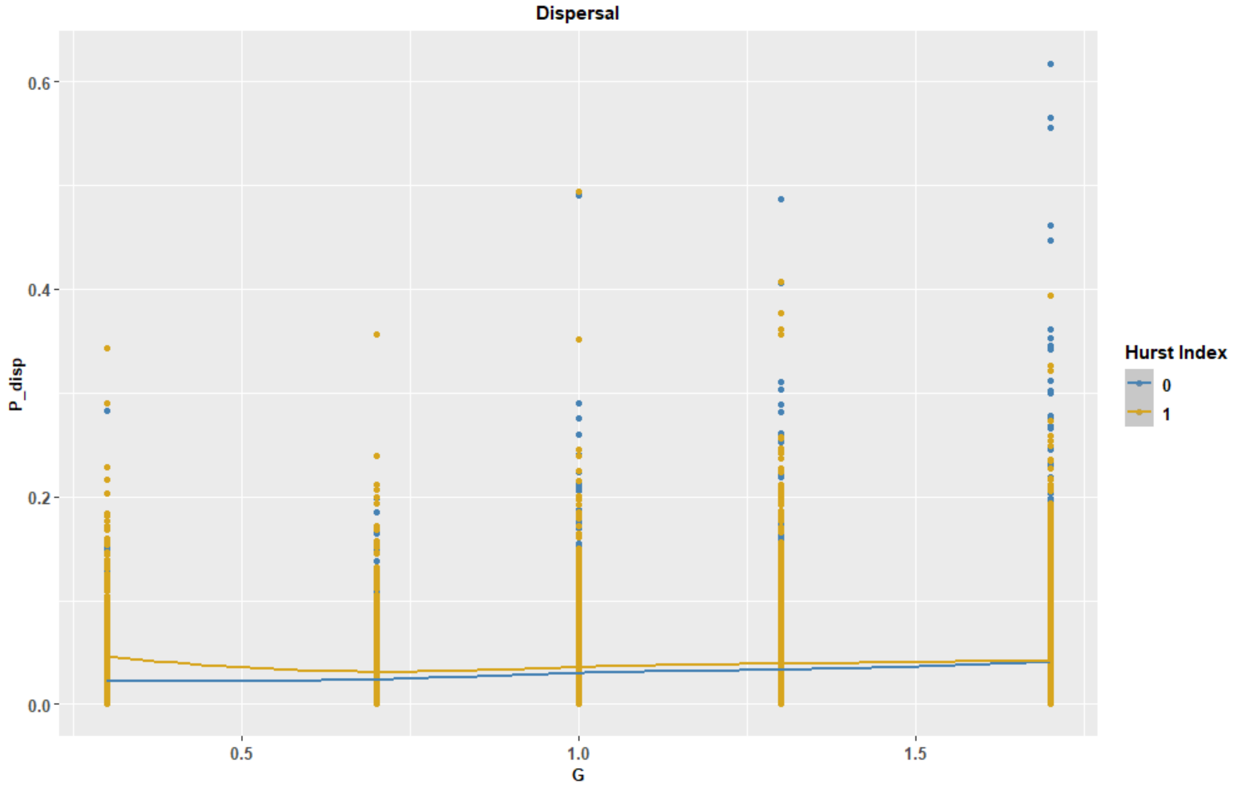

Fig. 2) Scatter plot of P_disp vs. G by Hurst index between G=0.3 and G=1.7 with GAM smoother with y axis restricted to 0-0.05

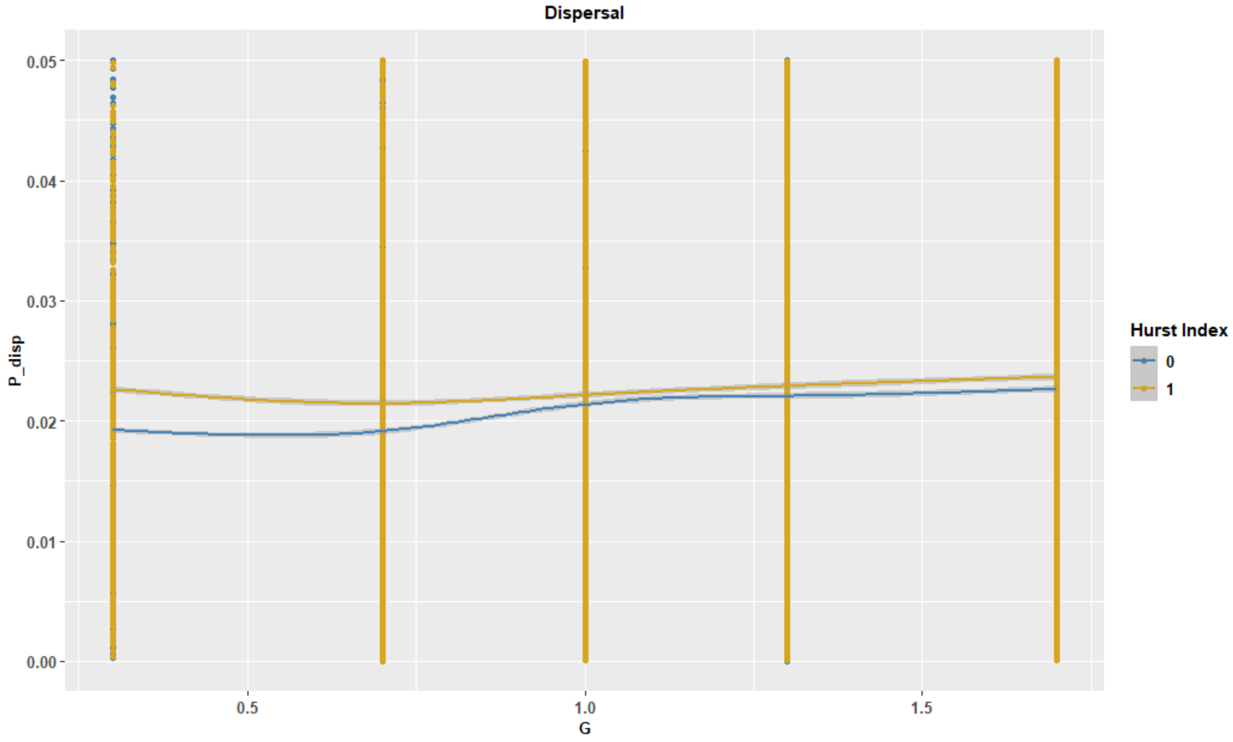

P_{disp} GAM summary for fig. 1) (via MGCV)

Family: gaussian
Link function: log

Formula:
P_disp ~ s(G, k = 5, by = Hurst_index)

Parametric coefficients:
Estimate Std. Error t value Pr(>|t|)
(Intercept) -3.50853 0.00506 -693.5 <2e-16 ***

Signif. codes: 0 '***' 0.001 '**' 0.01 '*' 0.05 '.' 0.1 ' ' 1

Approximate significance of smooth terms:
edf Ref.df F p-value
s(G):Hurst_index 4.971 4.999 626.2 <2e-16 ***

Signif. codes: 0 '***' 0.001 '**' 0.01 '*' 0.05 '.' 0.1 ' ' 1

R-sq.(adj) = 0.0283 Deviance explained = 2.83%
-REML = -1.9583e+05 Scale est. = 0.0011415 n = 99477

Fig. 3) ANOVA for log(P_disp) for scenarios G=0.3 through G=1.7

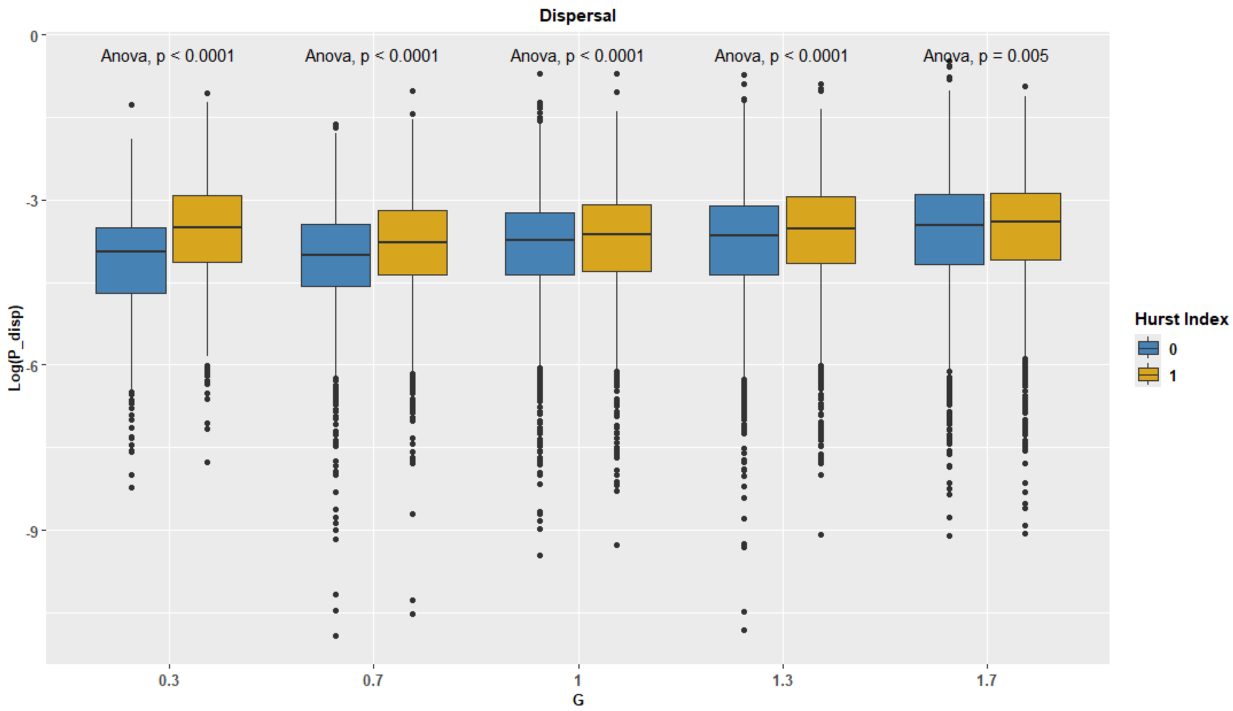

P_{global} (G≥0.1) GAM and ANOVA

Fig. 4) P_global vs. G by Hurst index between G=0.1 and G=1.7 with GAM smoother

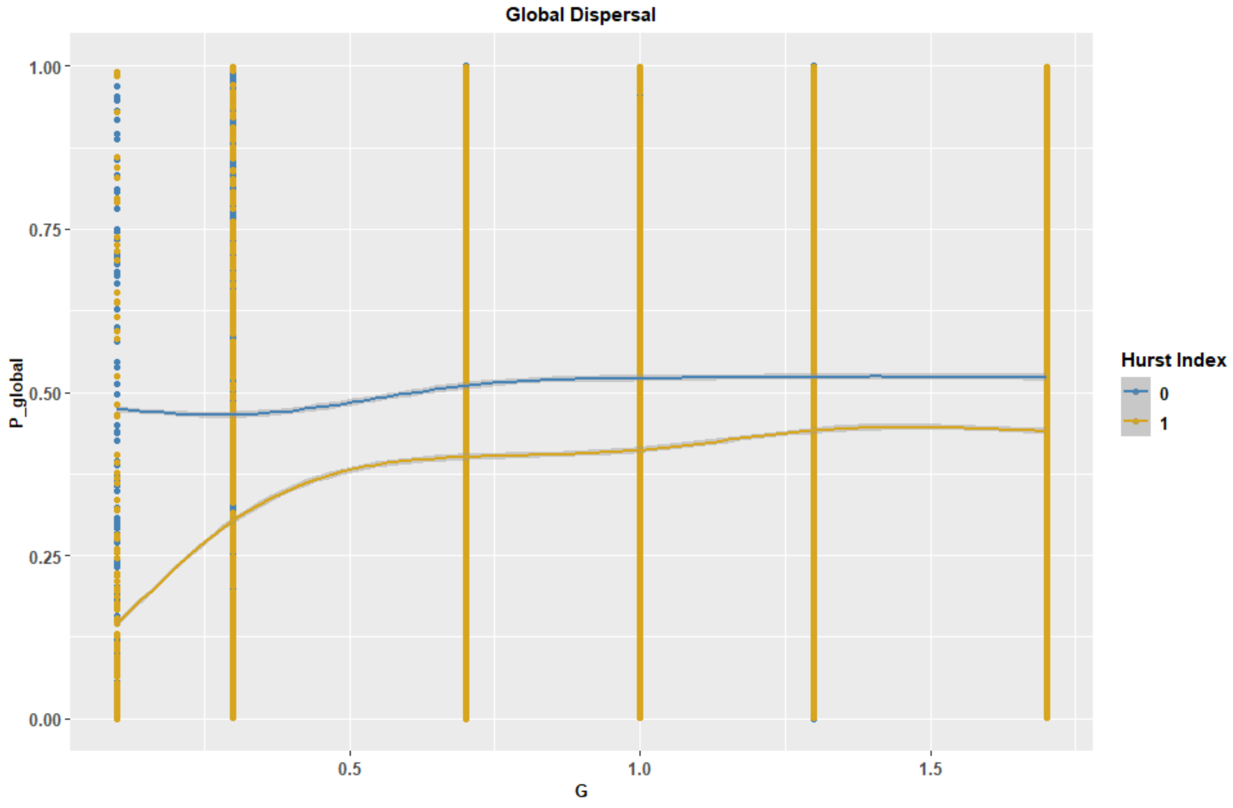

Fig. 5) ANOVA for P_global

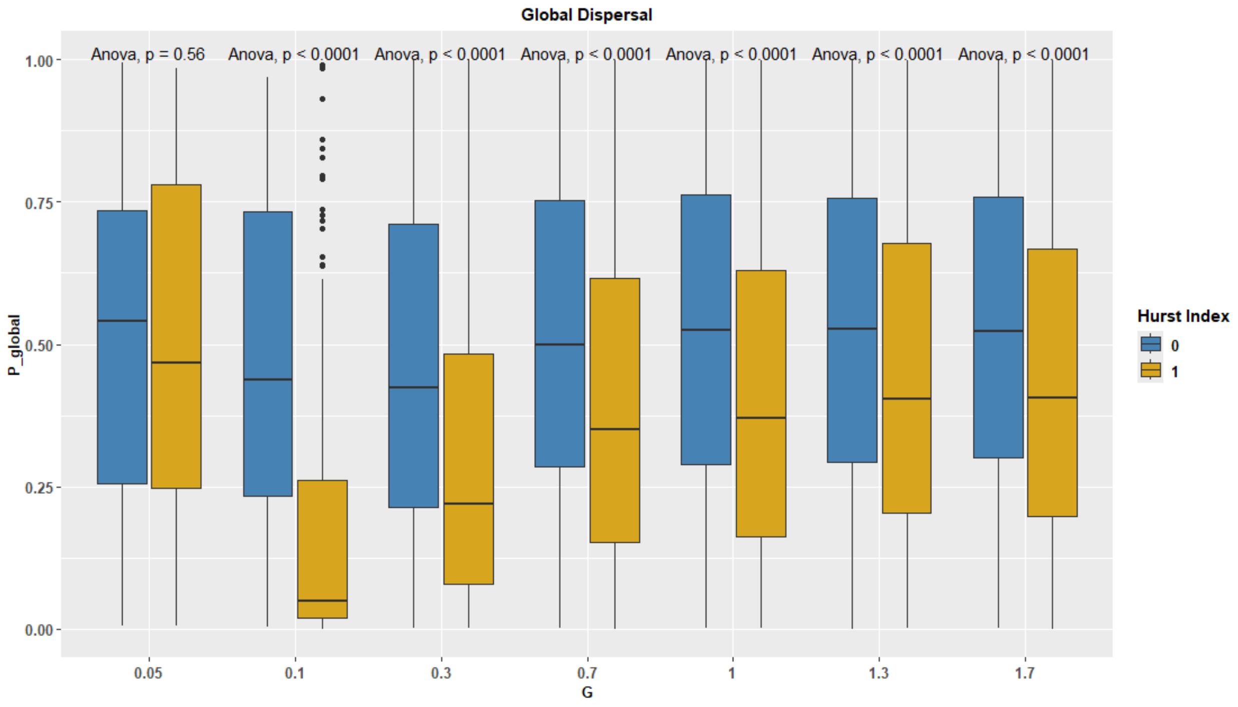

P_{global} GAM summary (via MGCV)

```
Family: gaussian
Link function: identity

Formula:
P_global ~ s(G, k = 6, by = Hurst_index)

Parametric coefficients:
            Estimate Std. Error t value Pr(>|t|)
(Intercept) 0.502929   0.001115   451.2   <2e-16 ***
---
Signif. codes:  0 ‘***’ 0.001 ‘**’ 0.01 ‘*’ 0.05 ‘.’ 0.1 ‘ ’ 1

Approximate significance of smooth terms:
                      edf Ref.df       F p-value
s(G):Hurst_index 5.969          6 2938  <2e-16 ***
---
Signif. codes:  0 ‘***’ 0.001 ‘**’ 0.01 ‘*’ 0.05 ‘.’ 0.1 ‘ ’ 1

R-sq.(adj) =  0.129   Deviance explained = 12.9%
-REML =  14194  Scale est. = 0.074219  n = 119477
```
